# Thinking OutsideTheBox - Designing Smart Things with Autistic Children

**DOI:** 10.1080/10447318.2018.1550177

**Published:** 2018-11-29

**Authors:** Christopher Frauenberger, Katta Spiel, Julia Makhaeva

**Affiliations:** HCI Group, Institute for Visual Computing & Human-Centered Technology, TU Wien, Vienna, Austria

## Abstract

This article offers a synopsis of and a critical reflection on the research project OutsideTheBox Rethinking Assistive Technology with Autistic Children. The aim of the 3-year project was to develop digital technology that would holistically respond to the complex life-worlds of autistic children, affording positive experiences that they could share with others. Through a series of long-term participatory design processes, smart objects were developed individually with nine children employing a wide range of different methods (e.g., Co-operative Inquiry, Future Workshops, Fictional Inquiry, Magic Workshops, Drama and Making & Digital Fabrication). In this article are presented the cases of all children worked with and tie them together by a critical reflection across them. The discussion offers insights along three main themes: we a) substantiate the argument for a theoretical shift in conceptualizing roles for technology in the lives of disabled people, b) discuss our methodological contributions in participatory design processes and c) propose alternative, participatory approaches to evaluate outcomes.

## 1. Introduction

There has been a great number of research efforts into the design and development of digital technologies for autistic^1^ people. Commonly, this is justified by the apparent tendency of autistic people to positively engage with technology as they perceive it as a safe and predictable medium that lends itself to their preferred interaction style (c.f. Silver & Oakes, 2001). However, there is little explicit discussion about the implicit agendas of these technological efforts. That is, while there is a lot of work in this area, there is less critical engagement with the underlying intentions and preferred outcomes. Most work is driven by the intention to assist, support or mitigate presumed deficits of autistic people and thereby often unwillingly or unintentionally pursue a normative agenda of “fixing people.” In their survey of the field, Kientz, Goodwin, Hayes, and Abowd (2013) state that most work included implicitly invokes the medical model of disability and, hence, focus on the “physical or functional limitations a person might exhibit” (p10).

Making presumed deficits the starting point for envisioning technology not only limits the possible design space, but it also rises ethical and moral questions about our understanding of disability in society. This article, then, describes the experiences our research team has made in the course of a research project that sought to explore alternative ways to think about technology for autistic people. With “OutsideTheBox Rethinking Assistive Technology with Autistic Children,” we set out to demonstrate that technologies can play alternative roles in the lives of autistic children and be meaningful, rather than narrowly assistive. We tasked ourselves to develop technology that would afford positive experiences for an individual child and would support the sharing of these experiences with others.

A central argument in this project has been that such technologies can only be designed *with* autistic children. OutsideTheBox has aimed to set up working relationships with autistic children in ways that would allow them to drive a design process with their ideas and desires that go beyond them being autistic, but were born out of their understanding of the world. Instead of reducing a child to their diagnosis, we aimed to holistically engage each child in a creative, yearlong process that would result in their very own, smart thing a ubiquitous computing artifact that affords meaningful interaction. Over the course of the project, we worked with nine children and involved them in the envisioning, designing, building and evaluating of digital technology.

The project aimed to make contributions in three areas: firstly, the argument to rethink the possible roles of technologies in the lives of autistic children requires us to develop a novel theoretical framework, connecting the critical perspectives emerging from Disability Studies and Science and Technology Studies with the paradigms of Design Research and Human–Computer Interaction (HCI). Secondly, methodological innovation is required to create environments and processes in which autistic children can meaningfully collaborate with designers in cocreating technology. Thirdly, new ways of evaluating the experience with the resulting technology are needed what does it mean to afford positive experiences with technologies in the context of autistic children and how can we assess these?

In the past 3 years, we have published successfully on all three of these areas. This article presents the overarching synopsis of our experiences and insights, aiming to make the whole bigger as the sum by connecting the dots and engaging in a critical reflection on how far we came in “rethinking assistive technologies for autistic children” and where we encountered new challenges which will be shaping our future work. This synopsis will stem from reporting on all our collaborations as case studies over which we subsequently reflect on through the three lenses: theoretical framing, process and outcomes.

## 2. The OutsideTheBox project

The project started June 2014 and ran for 3 years. Beyond the Principle Investigator, two PhD students worked full-time on OutsideTheBox. Our main objective was, to explore ways by which autistic children, aged 6–8 years, can lead the design processes of digital technologies to create smart objects that would be meaningful to them, are embedded in their life-worlds and allowed them to share the experiences they would make with that technology with others. We chose ubiquitous computing (UbiComp) as a versatile technological opportunity space that would allow us to explore a wide range of technological artifacts. Our hypothesis was that facilitating a child-led exploration of this UbiComp design space leads to novel applications for autistic children that emphasize positive experiences and wellbeing while providing appropriate levels of support and intervention.

Consequently, Participatory Design (PD) was at the very heart of OutsideTheBox. Over the duration of the project engaged children in three overall cycles, each spanning across a whole school year with the goal to implement different participatory methods to conceptually map out viable approaches to meaningfully involve these children.

Every engagement followed the same blueprint: after recruitment, we conducted a Contextual Inquiry (Holtzblatt & Jones, 1993), which involved interviews with carers, teachers and children, observations and probes. Subsequently, we engaged the child in design work, implementing one of six participatory methods which were chosen to cover a wide range of participatory styles. These six methods were: Co-operative Inquiry (Druin, 1999), Future Workshops (Vavoula & Sharples, 2007), Fictional Inquiry (Dindler & Iversen, 2007), Magic Workshops (Kuniavsky, 2007), Drama (Brandt & Grunnet, 2000) and Making & Digital Fabrication (Frauenberger & Posch, 2014). After an artifact was created, an evaluation phase would follow.

As we will report on below, the individual processes differed substantially; the overall framing and the design brief, however, were the same for all collaborations. Our goal to explore meaningful roles of technology in the lives of children and how they can support them in sharing positive experiences was precisely the overarching intent and purpose that we communicated to children.

**Figure 1.**
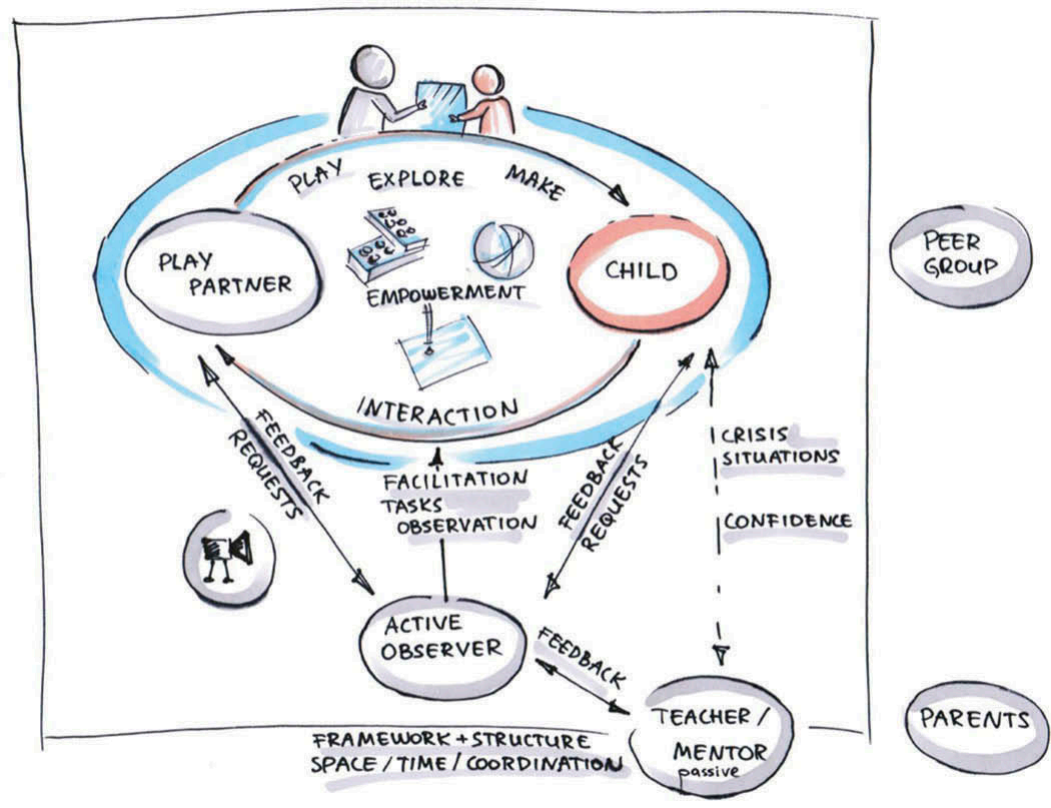
Roles and flow of interaction between play partner, child and active observer.

Access to participants was organized through the mentoring scheme of the local education department of the Viennese government. Informed consent was obtained and extensive information was provided to children, parents and teacher. We also established an ethics monitoring system that would provide safeguards for unforeseen developments or other events during our collaboration (compare Frauenberger, Rauhala, & Fitzpatrick, 2017). Sessions with children took place roughly fortnightly in separate rooms of their usual school environment or on the premises of our university. During the sessions two researchers were present and all sessions were video-taped. Inspired by the work of Feuser (2002), we developed designated roles for the researchers: the Play Partner teams up with the child and unconditionally supports the child with their skills. The Active Observer facilitates the session, sets the agenda, keeps the time, provides the materials, gives feedback and structures the session. This role is more detached from the actual design and the power difference created between the two adults (the Play Partner is supposed to follow the instructions by the Active Observer), results in the child teaming up with their Play Partner more easily. Figure 1 provides an overview of roles and interaction flows.

## 3. Case studies

The accounts below are based on a number of data sources to be able to paint a rich and detailed picture of our collaborations. We conducted semi-structured interviews with parents, teachers, mentors and other relevant carers before and after we engaged the children. All sessions with children were recorded on video and researchers kept a reflective research diary in which both perspectives, from the Active Observer and the Play Partner were brought together. Additional data was collected in the form of photographs taken by researchers and children, and actual artifacts created in the workshop sessions.

### 3.1. Overview

Table 1 provides an overview over the participants, their age, the diagnosis to the detail it was shared with us in the initial Contextual Inquiry phase, the name of their objects and the number of overall sessions. The children’s names have been changed to protect their identity.

**Table 1.** Research partners in the first year of OutsideTheBox together with age, diagnosis, design method used, name of the finished object and number of meetings; FW: Future Workshops, CI: Co-Operative Inquiry; MD: Makers & Drama; AS: Asperger Syndrome.

Name (Age)	Diagnosis	Method	Object name	#
Andy (8)	Autism	CI	ProDraw	10
Blaine (6)	AS	CI	ThinkM	14
Claude (6)	AS	FW	Adaja	13
Dean (8)	Autism	FW	DSmart	14
Quentin (9)	AS	Makers	Sound Boxes	15
Mia (9)	Autism	Drama	RattleC	17
Yvan (8) & Hank (6)	Autism	MD	TimeM	20
Oliver (6)	Autism	MD	Öxe	19

While each of the cases below describes a unique and situated process, we structure the accounts in similar ways: first, we situate the account by describing the child and the context in which we worked. Then, we describe the methodological approach and how this shaped the design process from ideation (exploring the design space), to concept (narrowing down), to implementation (realization). The final concept and object is subsequently described and the intended interactive experience. Finally, we report on the how these objects arrived in the life-worlds of these children and their first experiences with them.

### 3.2. Navigating needs with Andy

Andy was shy and did not like meeting new people. It took him several sessions to feel safe enough to directly interact with us. Even then, there was a pattern for each meeting: during the first 10 minutes or so, he refused to work with us. We had to rebuild our relationship anew each time for him to be able to trust as. While he clearly signaled at the start of each session that he did not want to work with us, we decided to continue with our collaboration, because after each session he would run to his teacher and explain how this was the best session so far and that he is so happy about what he did – even though he did not like using words and found it even harder to talk about his emotions.

**Figure 2.**
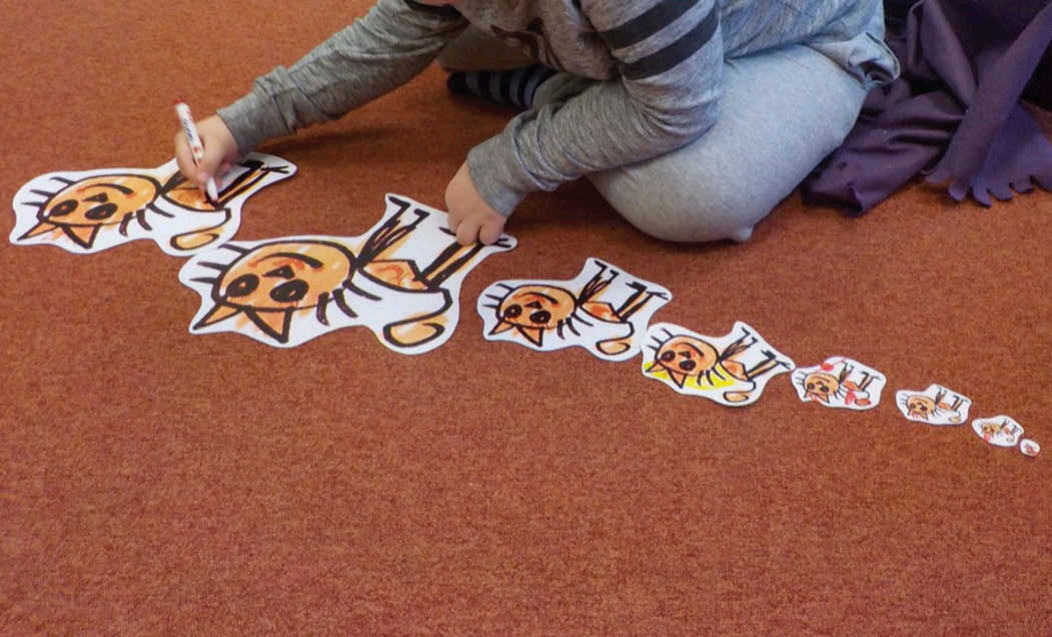
Andy’s cat at different scales.

We used Co-operative Inquiry (Druin, 1999), because it offers a flexible design method to engage a child along their interests and abilities. As a starting point, we used a set of elaborate drawings he made in one of the initial sessions. We explored them along different scales of size and observed his interactions with them (see Figure 2). He augmented the cats with little attributes which gave them different characters and personas. There was a princess cat, a grandpa cat and so on.

For his object, we were inspired by his incredible drawing skills, but were also conscious that his drawing isolated him from others. Thus we aimed to design an artifact that would build on his strengths, while allowing him to share his positive experience in ways he would feel safe and in control of the situation. We started to design a concept for an interactive drawing surface with which he could animate his drawings and project them onto a wall. Both private enjoyment and public sharing can be mediated through the object.

**Figure 3.**
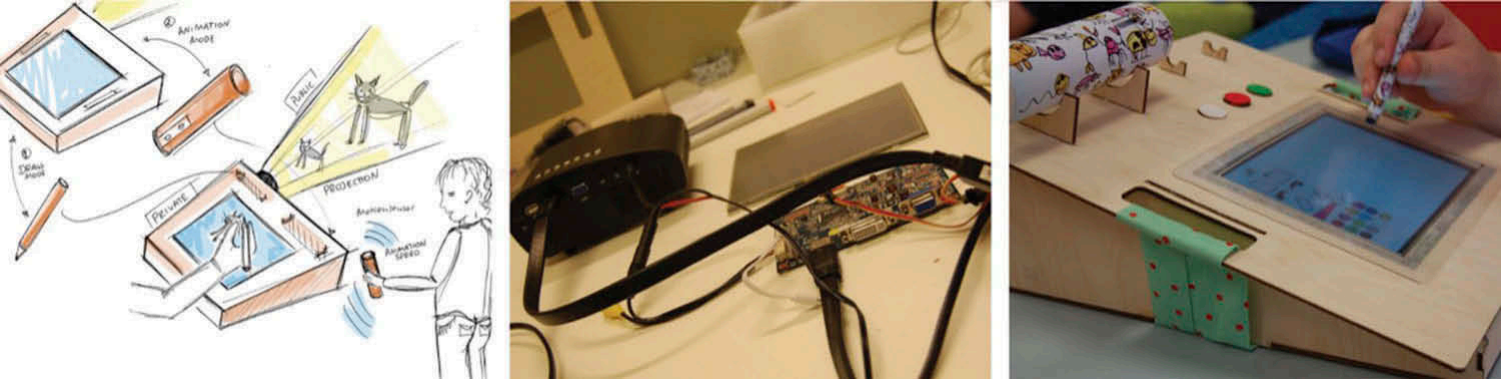
ProDraw showing self-created animations with embodied control.

In its final iteration, ProDraw (see Figure 3) consists of a touch surface that can switch between a drawing mode and an animation mode. Pictures drawn and saved in the drawing mode are automatically grouped for later animation. In the animation mode, a folder is chosen and the animation loops through the pictures in that folder to create the animation – quite like a flip book works. The speed of the animation is determined by the sensor data received from the Wii Remote Controller. The faster the controller is shaken, the faster the animation plays.

While the drawing mode of ProDraw does follow fairly paradigmatic standards of interaction with a touch surface, the animation mode forces Andy to take a step back and interact with the technology using his own body. The drawing experience is more private than the animation experience, which is addressed toward a larger space including a potential surrounding audience. While it is technically possible to always project what happens on the touch surface or to just turn the projector off during animation, having the animation react to the input of the Wii and how fast it is shaken, lets Andy literally step away from the technology and open himself up to others. This gives him full embodied control over his sharing experience. It opens up new spaces for interaction between himself and others that would not be possible by a static or non-embodied mode of interaction.

Andy presented ProDraw in front of his class and earned praise and envy – according to his teacher for the first time since he entered school. He was acknowledged for his skills rather than singled out for his perceived deficits. While he likes to share his finished drawings with others, he only rarely includes them in the creative process.

### 3.3. Engaging the environment with Blaine

Blaine engages animatedly in verbal discussions about his favorite topics – science, technology and inventions – but is easily overwhelmed by demands of social interaction. Repeatedly, this leads to difficult situations in class with his classmates or teachers.

From the start, Blaine identified himself as a researcher and scientist. Therefore, our working space was framed as a research lab, which he divided into designated areas for brainstorming and prototyping. We initially enquired into his interests through drawing activities and by discussing objects he liked.

Reinterpreting Future Workshops (Vavoula & Sharples, 2007) as our codesign method, we started to investigate current tools for research, before projecting them into future scenarios. This fed into his strong interest in science and provided a starting point for creative explorations. Blaine developed initial ideas based on his experience in class where he claimed he could not remember social situations in which he became very aggravated. He imagined tools that would allow him to conduct research into these situations and to find out what was going on. Consequently, he devised a machine to better concentrate with (Thinking Cap) and a machine to remind himself of past events (Remembering Machine). In the course of our work, the two merged into one.

**Figure 4.**
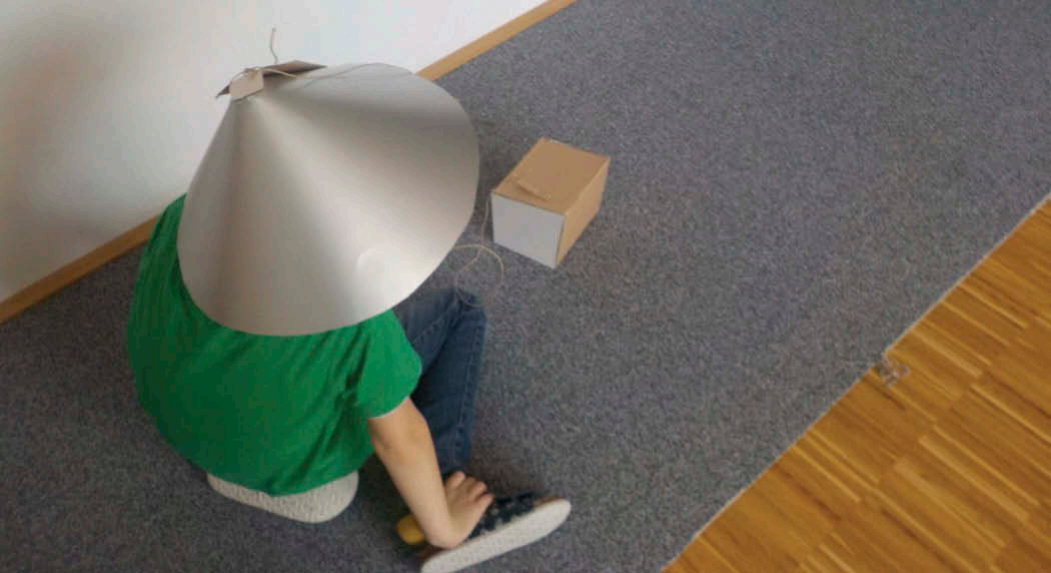
Playing out a use scenario with the second low-fidelity prototype of the thinking machine.

Initial paper prototypes allowed Blaine to play with forms and sizes of objects (Figure 4). He also specified certain interaction modi (e.g., data transmission had to be wireless and directed to a certain screen device). Collaboratively, we envisioned ThinkM – short for “Thinking Machine” – as a device that would allow him to capture and reflect on difficult social situations after the fact. Building on this, we decided to introduce Blaine to the possibility of using a pulse sensor and including this data in the visualization of captured events. Blaine quickly linked the pulse data with his emotional state through self-paced experiments.

**Figure 5.**
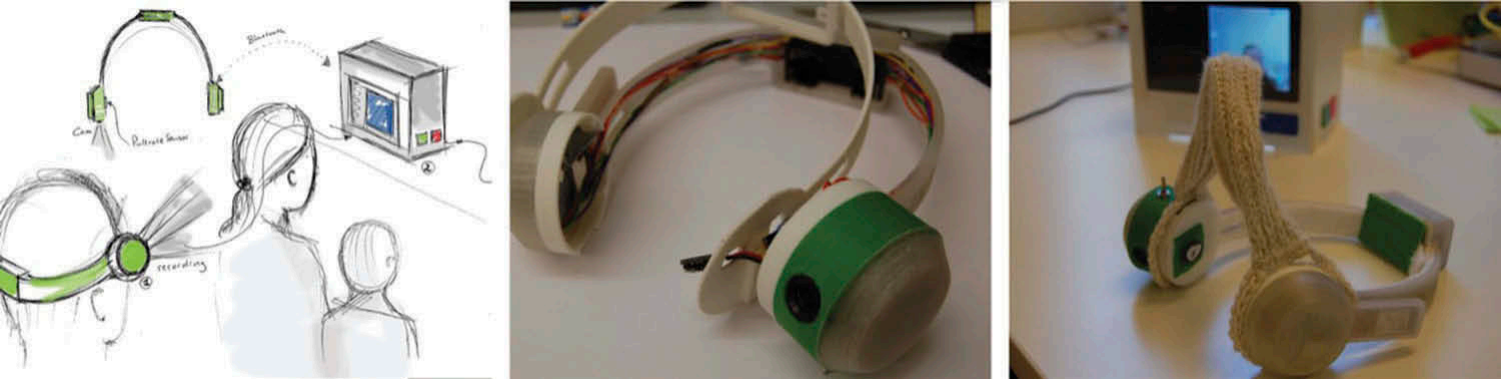
ThinkM embodied reflection of situations where the own body fails.

ThinkM in its final version consists of a wearable device in the shape of headphone headphones and a base station (see Figure 5). The camera is at the eye level of the wearer and records an image for every 10 s as soon as the device is put on. The pulse sensor is located on the inside of the headband. When the base station and the headphones are switched on and in each other’s vicinity, pictures and pulse data are automatically transferred. Over time, ThinkM loses some of its memory (i.e., older pictures) in order to mimic the behavior of a human brain – an analogy, Blaine introduced himself. After a week, half of the pictures in a folder are marked deleted, after another week, only a quarter of the pictures remain and so on. This feature also allowed us to address the privacy concerns that came up during discussions with Blaine, not only with respect to his own privacy, but also that of others. Additionally, we ensured that the data never left the system.

Next to being a stylish enhancement, ThinkM gives back control that was lost in certain situations. It provides Blaine with a way to make sense of them and helps him to reflect on his own behavior in a mode he understands as a “scientific” inquiry.

While it was not possible to present Blaine’s invention in front of his whole class, he unpacked the final prototype in front of his special educations teacher, an individual therapist and one parent. They praised him for his invention and he explained in detail how the different parts work together and what they do. He stated: “I invented this and you built it,” which indicates that he felt ownership of the design, but less so part of creating the actualized machine.

### 3.4. Investigating interests with Claude

**Figure 6.**
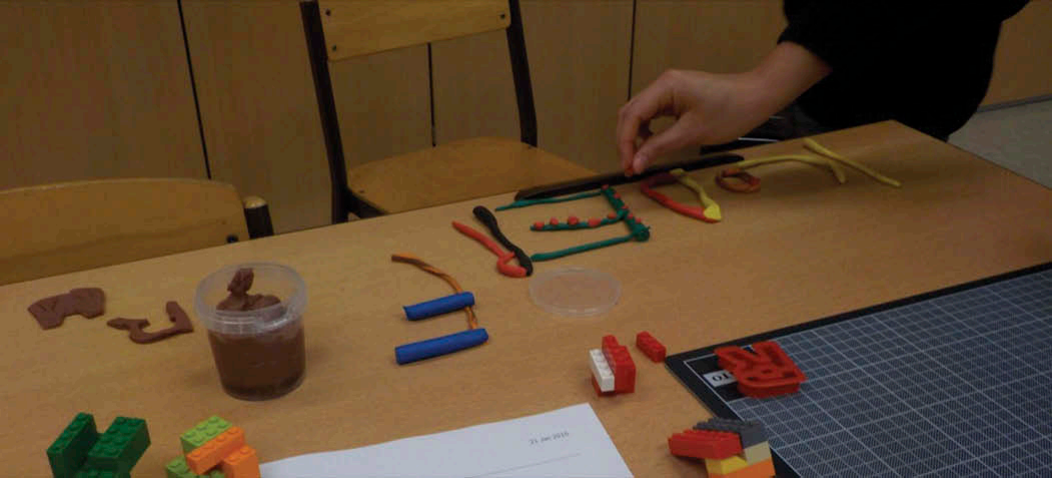
Different materials we used with Claude during the ideation and conceptualization phases bricks and modeling phase.

Using Co-operative Inquiry (Druin, 1999) we determined that an object for Claude would have to offer flexibility to continually capture Claude’s attention in various contexts. After trying different materials to understand more about the collaboration (some of which are depicted in Figure 6), we explored his use of a digital camera, electrical components for a smart car and his interaction with hidden letters in pictures. However, nothing seemed to go beyond a short-lived interest. Finally, when he interacted with a Kinect, we found that he was interested in exploring his surroundings, if they were represented through visually intriguing effects. After that, we decided that Adaja should visualize surrounding sounds and be a shareable device for exploration with peers.

**Figure 7.**
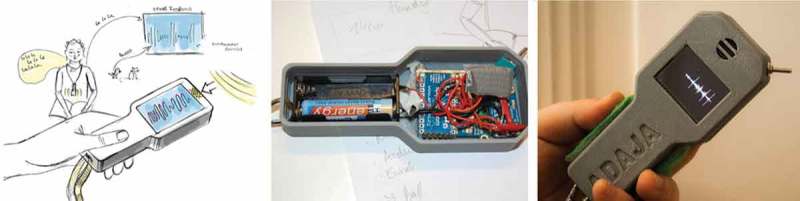
Adaja the ambient companion for exploring sounds of self and others.

We then experimented with different forms of visual representations of sounds on variable display sizes using wall projection, smartphones and bracelets. We noticed, that Claude preferred to interact with the prototypes in an ambient manner to calm himself. The final object, called Adaja (see Figure 7), is arranged into a wearable ambient device that constantly updates its screen according to the intensity of noise it records.

With Adaja, Claude can explore the sounds of his environment. He can share the display exhibiting a visualization of the loudness of incoming sounds with others or tilt it so that he alone can interact with it. Whenever a certain threshold is reached, Adaja displays the words “too loud,” to support him regulating his voice. In classroom situations, we could observe how the affordances of individual and social interaction led peers to ask Claude to share his experiences with Adaja, which opened up even more opportunities for interaction. However, even in its final realization, Adaja was ultimately only briefly interesting to Claude. He returned the object during our last meeting.

### 3.5. Moving pictures with Dean

**Figure 8.**
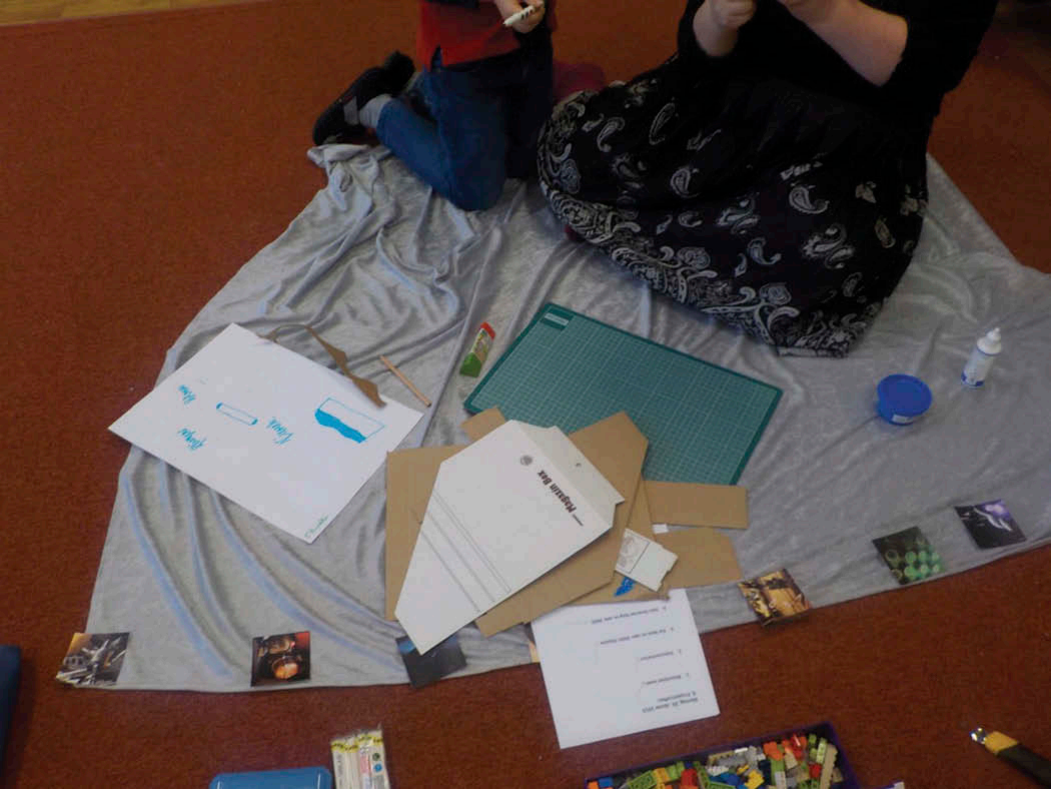
A magic silver carpet transporting us into the year 3000.

In our collaboration with Dean, we adapted Future Workshops (Vavoula & Sharples, 2007) with elements of Fictional Inquiry (Dindler & Iversen, 2007). We started by planning the second episode of his favorite film, “Brave,” set in the future. That made it possible for us to explore future everyday activities. For a stronger effect, we introduced a “magic” silver carpet (see Figure 8) that transported us to the year 3000. From these explorations, the fundamental concept of DSmart emerged which would combine watching trailers of upcoming movies and supporting Dean in telling his own stories by providing appropriate prompts and inspirations.

**Figure 9.**
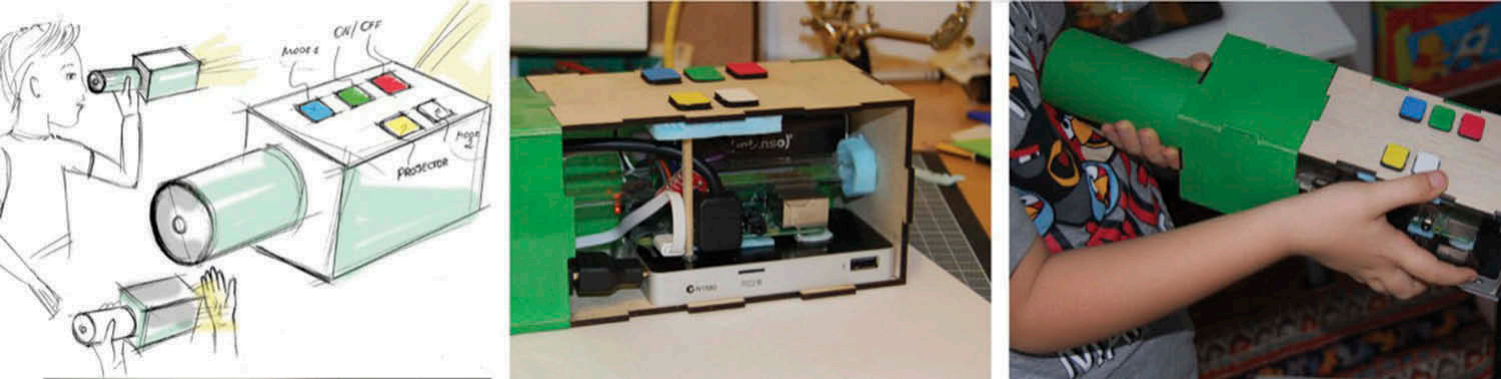
DSmart the smart companion for telling stories and investigating upcoming movies.

DSmart in its final iteration is a smart companion in the form of a kaleidoscope that not only informs about upcoming movies, but can also give prompts for storytelling (see Figure 9). It functions as a conduit between Dean and his environment, making the interaction more controllable and, hence, predictable. Dean can choose between the video mode displaying up to three movie trailers, one after the other, or the story mode, which shows up to 10 pictures of agents or backgrounds. When the limit of movies or pictures is reached, DSmart enforces a pause to avoid a narrow focus on single activities – as per Dean’s suggestion (!). He wanted his object to be able to do several things which are unrelated to each other. Accordingly, the story mode and the movie mode function independently from each other.

In observation of Dean’s interaction with the object, we could see that he showed *Reactive Embodiment*. He physically reacted to what happened with the object which on the other hand influenced how the object presented its content. For example, he investigated, how the story changes when pointing the projector on different surfaces, reacted gleefully to the change, involuntarily moving DSmart in his hands, creating a new picture on a different surface, which again made him explore more or react again.

### 3.6. Fabricating objects with Quentin

**Figure 10.**
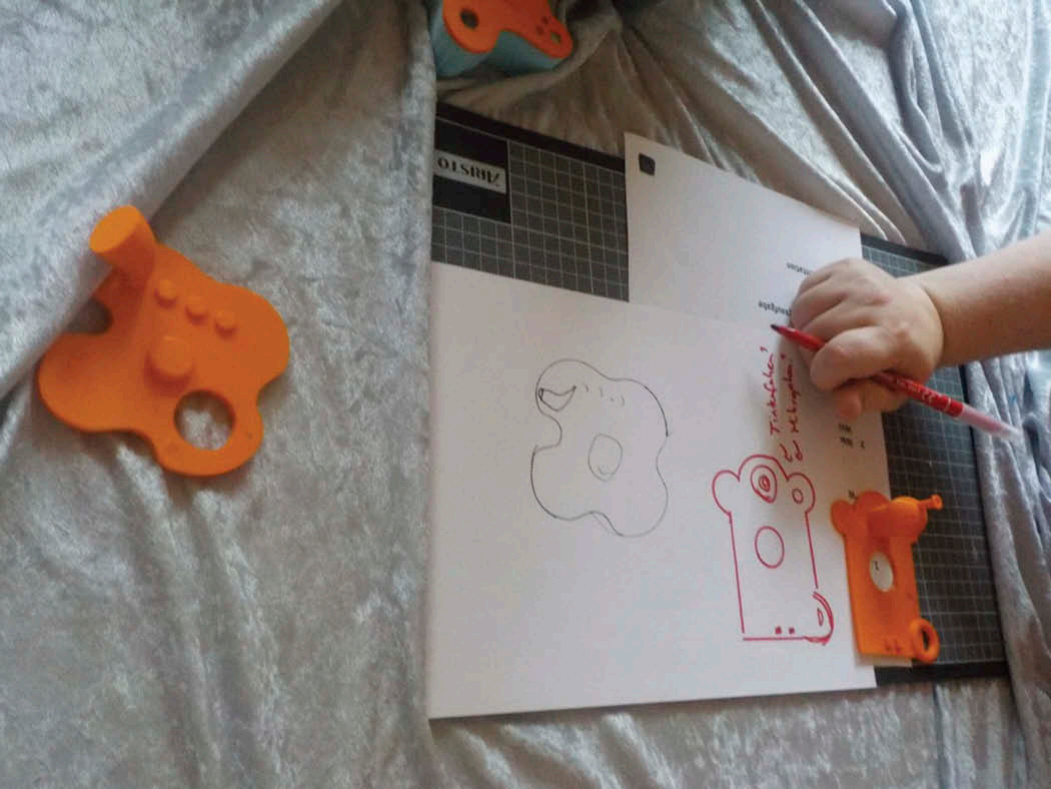
Experimental objects we used for inspiration.

**Figure 11.**
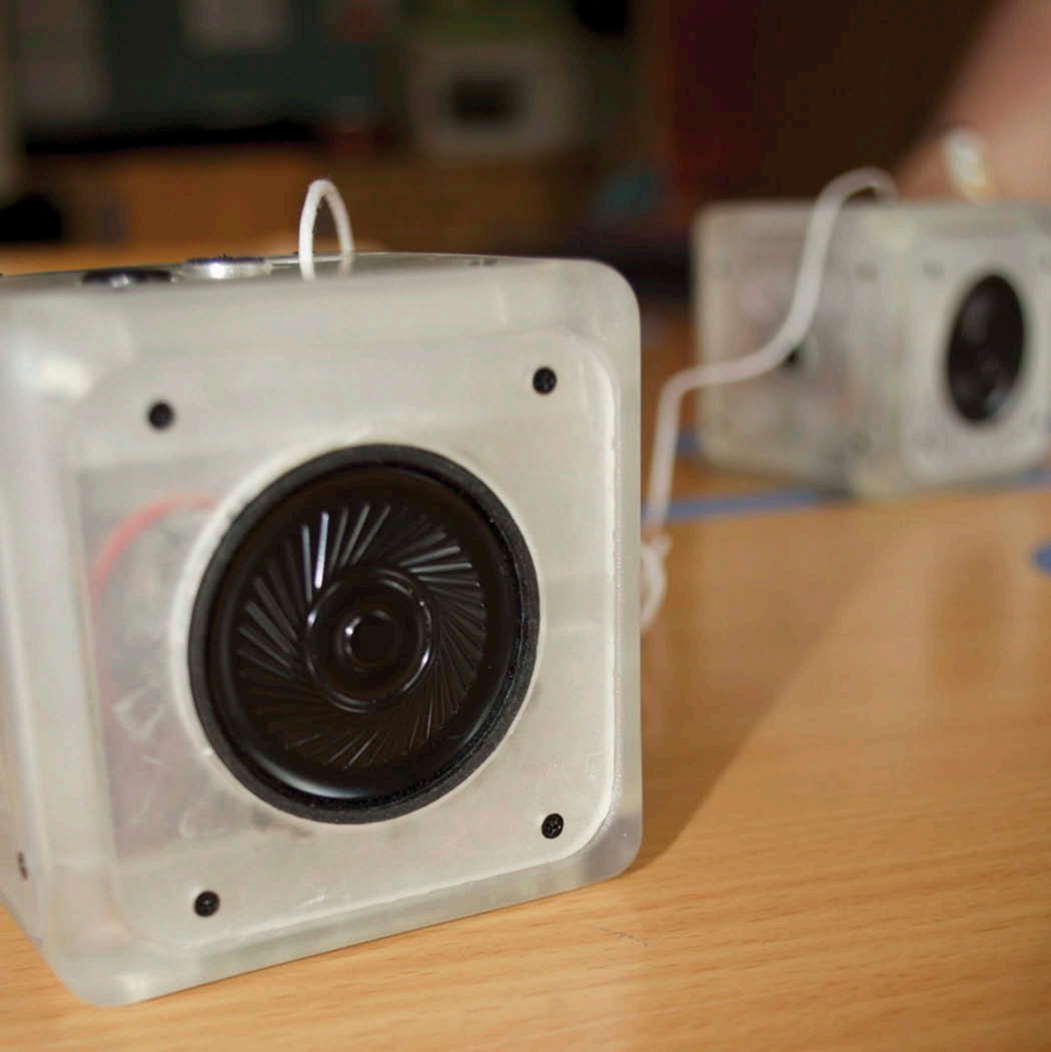
Sound Cubes – developed together with Quentin.

Quentin was very caught in the ideation of things he already knew or got to know via his science club. Hence, we also tried out experimental objects (see Figure 10) to tease out new ideas. On our design journey, two sessions were conducted at the university, where 3D-printers, a laser printer, a CNC machine and several smaller fabrication tools are available (following the concept of Digital Fabrication Frauenberger & Posch, 2014). Inspired by the potential of these machines and a prototype for exchanging sound messages from a different research project,^2^ we decided to pursue a similar object and developed the Sound Cubes (Figure 11).

Of all children, Quentin was involved the most in actually creating his final object. He was very enthusiastic in using a soldering iron to connect the different parts. However, it was hard for him to understand, that the object was sufficiently complex that there were further steps to do in a different session. While he indicated no issues in working with a set of prefabricated elements, he wanted each session to come to a conclusion in terms of a finished object that he could take home. The fidelity of the object was then secondary.

The Sound Cubes were realized as a pair. However it is technically feasible to create additional cubes so that any cube could interact in the same way with any other. The cubes can record a sound message, replay it or transfer it to another cube by pressing them together. Every cube can also receive messages from any other and play these. Each side of the cube is dedicated to a different function: one for the speakers, one for the microphone and recording, one for message replay, one for receiving messages, one for dropping a message and finally, one to place the cube on.

### 3.7. Morning routines with Mia

**Figure 12.**
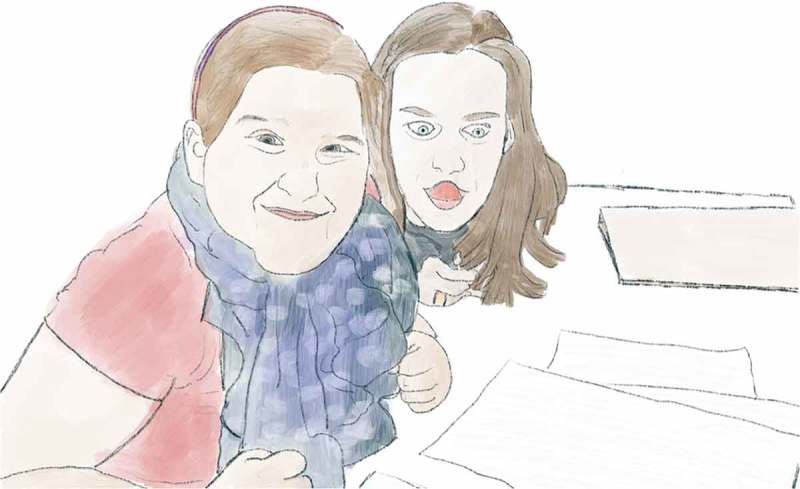
Mia and her play partner grimacing at the camera as ritualistic part of our routine.

Mia loves everything related to Super Mario games with Toad and Yoshi being her favorite characters. She also liked communicating with us under her terms. For example, in each of the sessions, we had to make a picture of Mia and her Play Partner grimacing into the camera (see Figure 12). This was part of a ritual we established early on in our collaboration. There were lots of opportunities for her to shape our interaction and whenever we brought something to the table, she consistently engaged with it and made it her own. That way, she reinterpreted methods we introduced and used them in interacting with others as well, when she felt this was appropriate. She had a strong sense of social rules or rather when she was breaking them. Hence, she sometimes let some of her toys speak for her. During our collaborations she used Yoshi, Bowser, Super Mario and later Link in order to communicate things she did not necessarily feel safe to express in her own voice.

Using the semantics of a Super Mario game world, we used theater methods (Sato & Salvador, 1999) and augmented them with playful elements to learn more about Mia’s life context (Spiel, Frauenberger, Makhaeva, & Kayali, 2016). We established that she finds getting up in the morning very irritating. She suggested that we create a cushion that wakes her up by vibrating next to her instead of the disturbing sound made by her then-current alarm clock.

**Figure 13.**
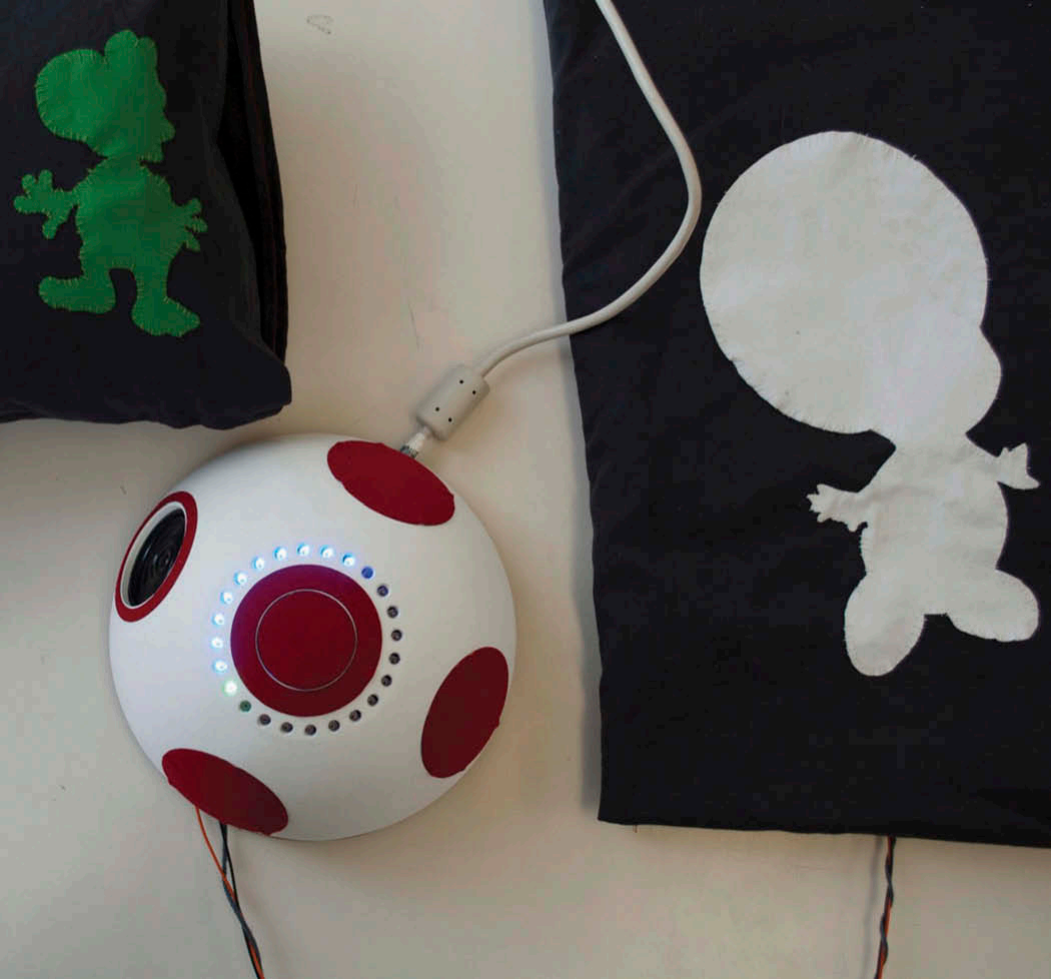
The rattle alarm system – Developed together with Mia – left: cushion, middle: alarm clock, right: pressure mat.

The resulting Rattle Alarm System (see Figure 13) consists of three parts. At the core, there is an alarm clock module – aesthetically modeled after Toad’s head – which displays the current time through blue lights. The alarm time can be set through a light touch on top of the module and is displayed with a green light. When the alarm goes off, the Super Mario theme song plays in an endless loop and the cushion vibrates. The alarm can only be turned off by getting up and stepping on the pressure mat. Shortly after the alarm is turned off, the clock plays a little melody – different each day – which sets the mood of the day as a stand-in for a horoscope. We made sure it always ends on a positive note even though a more mellow tone can be set before.

When the rattle cushion starts vibrating together with the engaging Super Mario theme song, Mia perceived this as a cheery person, waking her up with a gentle touch. Getting up itself also becomes embodied by having to stand on the map to turn of the alarm. There is no snooze functionality. Sharing becomes much more implicit in this context as the positive experiences with this technology would not be explicitly shared, but influence Mia’s interactions with others for the whole day.

### 3.8. Exploring (social) spaces with Yvan

Through Contextual Inquiry, we not only learnt more about Yvan’s core interests, but also how important his 5-year-old brother Hank is to him. During our ideation phases he constantly envisioned his brother to be present in potential use contexts. Yvan also talks at length about geography, planets and space travel whenever he could; not always considering whether his audience is actually interested in listening. He was focused on developing something that allowed him and his brother to explore faraway places – preferably in space even. Once we settled on the idea of a Time Machine, with which we could travel through time and space, we explored the actualization of this idea through means of Digital Fabrication (Frauenberger & Posch, 2014). We eventually decided that it would consist of two parts: an immersive light blanket and a navigation interface.

**Figure 14.**
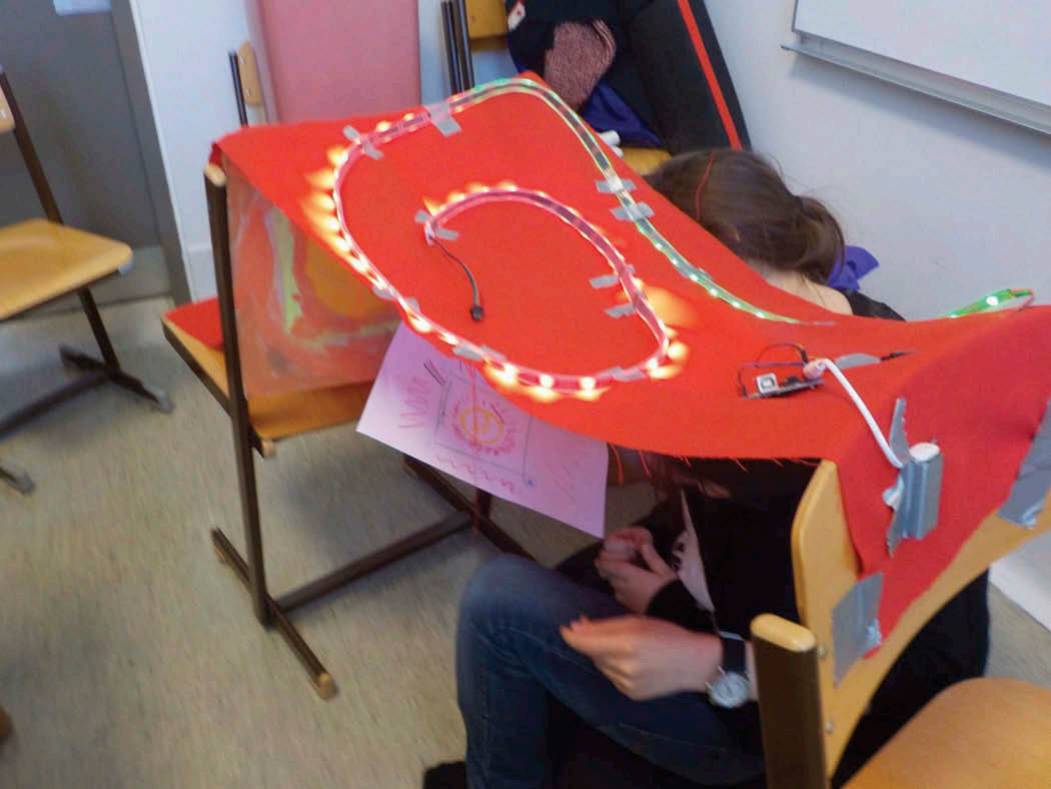
A mid-fidelity prototype of the time machine for use case exploration.

Yvan was quite enthusiastic when it came to prototyping his ideas. While other children had issues with conceptualizing cardboard prototypes as stand-ins for later, more finished objects, Yvan had no problem interacting with them as is (see Figure 14). He enthusiastically provided design critique and suggested alterations to the design that would improve it from his point of view.

**Figure 15.**
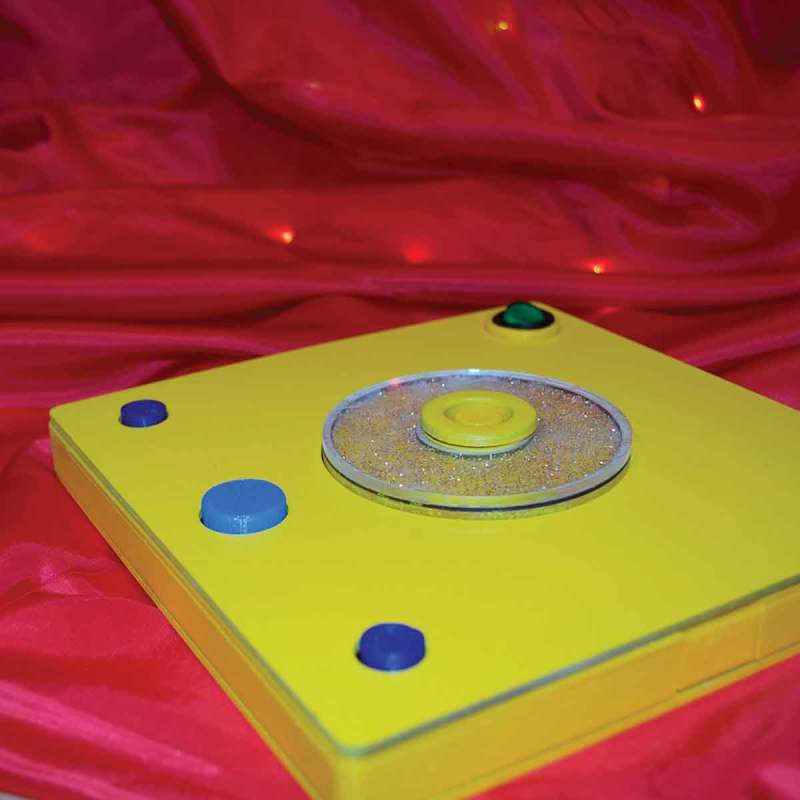
Time machine in the foreground, the navigation interface, in the background, the immersive light blanket.

While the technological parts of the Time Machine (see Figure 15) are relatively simple, the smartness of the technology emerges in use. Through the navigation interface, a user can control different light patterns on the blanket. They only become meaningful through the narrative established between users. Yvan then tells elaborate stories in which he travels to different planets at different points in time. Once he lands, he steps out of the machine and grabs different things in the environment, but gives them a different meaning, appropriate to the time and place he traveled to. This type of pretend play is notoriously difficult for autistic children (Jarrold, 2003). However, the Time Machine introduces just enough structure for Yvan to do so cooperatively. Another effect of the Time Machine is that it becomes a productive release for Yvan’s specialized knowledge that engages another person on equal terms. They experience the immersive space together and can both shape the narrative. The specialized knowledge becomes part of a joint adventure instead of a one-sided lecture.

### 3.9. Traveling through time with Yvan and Hank

**Figure 16.**
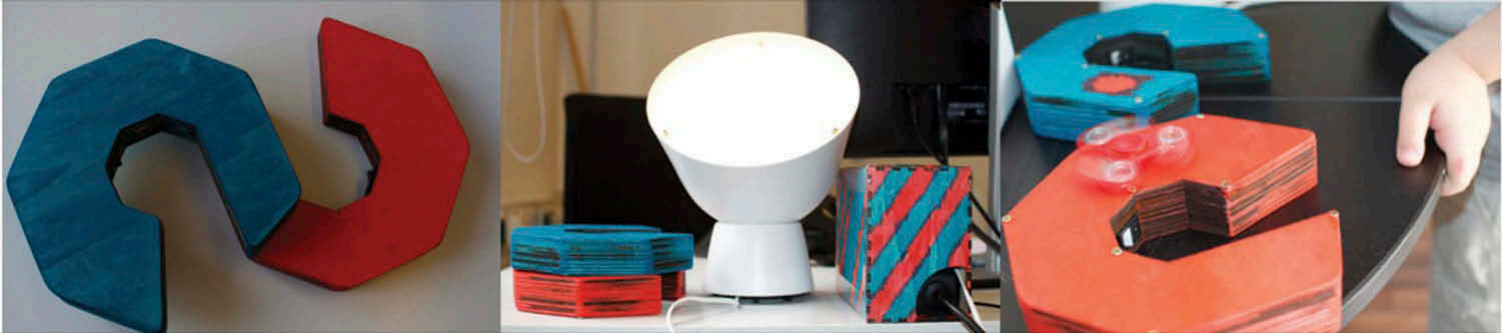
The time machine in its latest iteration. The two navigation elements, all elements and appropriation of the light through a fidget spinner.

After the first working prototype, we continued our collaboration, but included Yvan’s brother Hank. We noticed that Hank, being the younger brother of the two, often required additional support from either one of us. Conceptually, we sought to broaden the concept of time-travel for the brothers to resolve frequent conflicts over control. At the same time we were acutely aware that a new version needed to be more robust than the previous. Our goal was to design something that they could interact independently with, but provided incentives for collaboration and joint play. The final version of the time machine, seen in Figure 16, illuminates the whole room with a lamp and comes with two different, but similar controls. The intensity of the light changes with sound input, whereas the colors change along with the input coming from the gyroscope. When both navigation elements are put together, they activate a rainbow light show, where the lamp circles through several colors. Both children interpreted that as the travel part of their adventures.

### 3.10. Narrating numbers with Oliver

**Figure 17.**
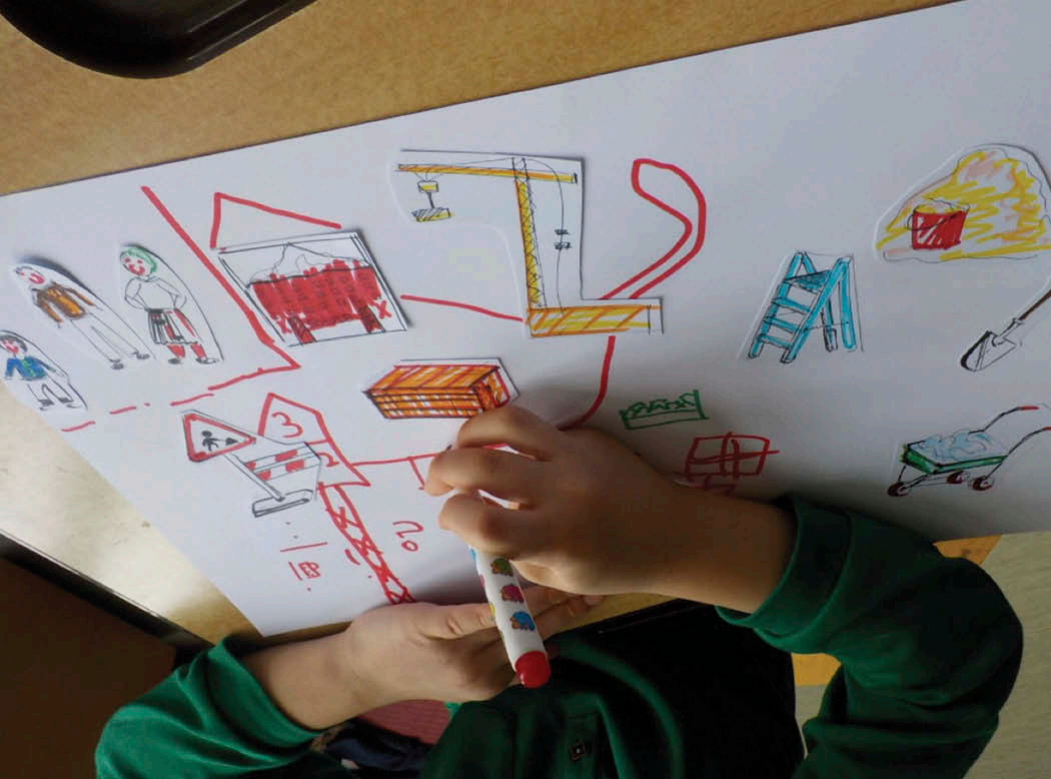
Oliver drawing around construction elements.

Oliver was very interested in building and construction work as well as elevators and drawing. Additionally, he developed a core interest into maths number games. We conducted a series of narrative-driven maker-workshops during which we investigated ideas and built prototypes hands-on (Frauenberger & Posch, 2014). We started by creating a “construction site” for his smart object. He could sort, alter and expand on construction elements and tell a story (see Figure 17). Incorporating mixed media, such as Lego or Plasticine was effortless for him.

Inspired by his interests in maths and narratives, we designed an interactive light table on which he could create images with tokens representing the alphabet, numbers or animals. While we determined the specifics of the functionality and aesthetics over the span of several sessions, we could also observe how he appropriated certain modes, such as moving the number games toward the table. This way, we could support these appropriations during development.

**Figure 18.**
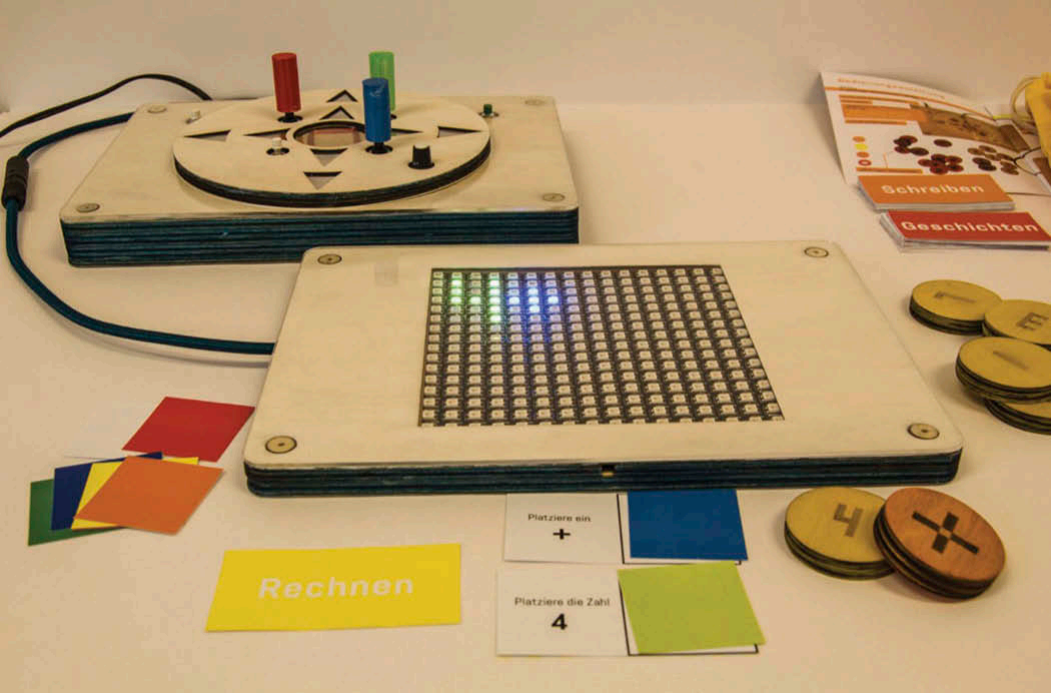
The light table in its final form with elements for math games, writing and storytelling.

As can be seen in Figure 18, the final object consists of two core elements: a table with an LED Matrix and a control table with which Oliver can move the drawings, animate them and undo previous steps. Additionally, there are tokens to create numbers, letters and animals. We also provided a set of preformulated games and tasks that can be solved individually or collaboratively.

The light table provides several options for solitary engagement or interactive opportunities with peers and other adults (teachers, parents). As the core expert, Oliver animatedly shows others how it works and what can be done with it and he also guides them through the interaction with the object. Being so familiar with it enables him to come up with ideas for further games and tasks. We were also able to use the object itself to evaluate our design process with Oliver as it is so open for all kinds of purposes.

## 4. Critical reflection

The above accounts tell the stories of collaborations with children that all resulted in the creation of a “smart” object. Despite having a common brief and similarities in methods or settings, the narratives are also very different and reflect the diversity in the group of children we worked with. Some of the resulting objects may seem mundane or just like another toy, but looking at the ways they were created and later used, or not, we believe that these case studies offer insights for rethinking the possible roles of technology in the lives of autistic children and the approach by which we can design them.

In the following sections, we aim to tie these experiences together and reflect the cases through three lenses, which correspond with the three areas that we had identified in the beginning as the three areas in which the project would make its main contributions.

### 4.1. Theoretical framing

The core premise of the project was to engage autistic children holistically (i.e., reframing the design of technology in this context by not taking the disability as the starting point). Mankoff, Hayes, and Kasnitz (2010) first made the argument that our conceptualization of disability determines the kinds of technologies we develop. They asserted that while a medical model is pragmatically useful, as it provides concrete requirements and evaluation criteria for the design of technology, it is also reductive and does not consider the many other ways in which technology can be meaningful for disabled people. We have built on this argument and in Frauenberger (2015) propose to adopt an interactional model that emphasizes that the disabled experience is multi-faceted, created by the inter-play of intrinsic and extrinsic factors. These include the biological differences or personal attitudes as well as socially created stereotypes, economic situation or accessibility issues. As a way of underpinning this, we have proposed critical realism as a philosophical framework that allows looking at the same reality (i.e., the disabled experiences) on different levels and from multiple perspectives.

There is no denying that many traditional assistive technologies, which focus directly on mitigating a functional limitation, have made a significant contribution and sometimes made a difference in the lives of disabled people. We would not want to diminish this contribution, but argue that a focus on assisting with functioning is only one part of what technologies could mean for disabled people.

The above cases demonstrate that designing technology from our perspective leads to outcomes that would have been inaccessible to traditional assistive technology approaches. While autism was part of each of the stories, we believe the outcomes are about more than the functional limitations of these children. For example, in Dean’s case, the object specifically supported him in his storytelling, challenging him in his repetitive thought patterns. However, the object also engages with Dean’s disabled experience on multiple levels. It spoke to his strong interest for a particular children’s movie, without locking him into this world, but connecting with his desire to share his stories with others in a safe way. The object was designed to be like a magic wand that provided a safety net in a socially risky situation. This is similar to Andy’s case, where his strategy to emotionally regulate by drawing was reframed into an activity that would not lock him into his own world, but became shareable. Our approach proved to be effective to engage the disabled experience of children on multiple levels to create meaningful artifacts.

However, this reframing and this change of perspective, also fundamentally challenges some of the foundations in the field. In conducting research and building knowledge, it requires us to embrace a different science paradigm. While a deficit-centered approach readily suggests a (post-)positivistic mode in which the effect can be objectively qualified, shifting our view to a holistic approach, as we have aimed for, makes such statements much more difficult, sometimes impossible. Design briefs turn into wicked problems (Rittel & Webber, 1973) which defy a definite formulation and for which no single best solution can be found. How, then can we learn from these cases and transfer our knowledge to other cases?

In this project we have mainly published on methods or theoretical frameworks, but rarely felt we could substantiate knowledge in the designs themselves. We have spoken about *how* and *why* to create these technologies, but not published guidelines for *what* should be built. While traditionally assistive technology research built up artifact knowledge, shifting our perspective did not allow us to do so. We would argue that this is not necessarily a fault, but a feature as it emphasizes the unique situatedness of each disabled experience. Various scholars in HCI have struggled with this problem, in particular within the concept of Design Research (see for example Gaver & Bowers, 2012; H¨o¨ok et al., 2015; Zimmerman, Stolterman, & Forlizzi, 2010), but little of that thinking has reached the field of assistive technologies. Possibly, because disability as a context is far more heterogeneous as designing in the mainstream and finding family resemblances or strong concepts is far more elusive. In this project, we have experimented with a variant of annotated portfolios which we called Design Exposès where we described design artifacts embedded in the process (Frauenberger, Makhaeva, & Spiel, 2016), but the main contribution remains procedural, rather than artifact-based.

As a consequence, we face the challenge of scaling. Critics of our approach might say, it is not surprising that we created meaningful objects for the children we worked with, given the amount of attention, time and resources we threw at them. But how would we design for well above 1% of the population, which is the approximate prevalence of autism^3^? While we argue that knowing *how* is a significant part of that puzzle, we have yet to find better answers to this question.

### 4.2. Process

In the planning of this project, our goal was to map out a range of participatory design methods that would embody our principle approach. While there exists guidance in the literature on who to adapt PD methods to working with disabled children (e.g., Benton & Johnson, 2015), the premise of a holistic approach required a more substantial reinterpretation. In the first year of the project, we largely adhered to following existing methods (Contextual Inquiry and Fictional Inquiry), but increasingly found ourselves working in different ways. In Frauenberger, Makhaeva, and Spiel (2017) we reflect on our use of methodological building blocks to tailor the process to the child and the individual context we worked in. Similar to a methods tool box, we found ourselves fluidly using and blending elements of different methods, not only in response to the context, but also considering the previous experiences made with each child. Based on our own expertise, the child’s background, the environment and prior results, we would design each upcoming session anew to move the process further. Such methodological choices are typically nontransparent, but with Frauenberger et al. (2017) we have attempted to provide a systematic way to arrive at a coherent string of design activities.

Two interconnected concepts have played a significant role in doing so: Creativity in autism and Handlungsspielraum. As we have worked with the children, we sought to strike a delicate balance between providing structures and freedoms to enable them to think creatively about technology. Structures were provided through known materials, routines as well as our own roles. Freedoms were opportunities for the child to leave their comfort zone and create something new, sometimes in the form of new materials or activities. We called the space that is created by structures and freedoms a *Handlungsspielraum*^4^ (Makhaeva, Frauenberger, & Spiel, 2016). This reflects out approach in all the cases described above. It has helped us to design individual sessions, but also more generally described the way we engaged children in a creative process, being careful not to overwhelm them. All our cases start with the child’s special interest which then is gradually and carefully modified to lead the child into a creative process. The concept corresponds to the idea of *flow* by Csikszentmihalyi (2009) that balances challenge with skills, while here we aim to balance structures and freedoms in design work with autistic children.

Illustrative examples from above include Mia’s case where the world of Super Mario provided a sense of safety and sameness, while we carefully hitchhiked it as a lens for looking at other aspects of her live, in this case her morning routine. It then allowed us to playfully explore possible roles of technology in this space. In Oliver’s case, his drawings, stories and fascination for lights led us to experiment with a light-table. Again, known concepts were gradually altered by introducing new aspects in order to allow the child to creatively play with them.

At this point, we also reflected more deeply on the question how we understand creativity in autism. While few studies have looked into the concept in relation to autism (e.g., Craig & Baron-Cohen, 1999), we sought to develop our own understanding that fits our theoretical framing. In Makhaeva et al. (under review), we explore different meanings of creativity and arrive at a conceptualization for our work, that is characterized by being situated, continuous, cumulative, experiential and embodied. In designing activities for children that enable them to unfold their creativity, this provides a valuable perspective, as it highlights the need for facilitating a range of design experiences that become the repertoire with which a child can be creative with. In most design cases above, we have used littleBits,^5^ for example, to expose children to the idea of causal relationships between sensors and outputs in technology. Further on, we brought children into the situation where they could build on these experiences to express their own ideas.

In terms of the fundamental research question of this project, if autistic children can be enabled to lead open design processes with their ideas, we are confidently answering yes. All our case studies have shown that their course and outcomes were significantly shaped by what children brought to the table. Our methodological work on the roles of researchers, innovation in methods of participation, systematic ways of blending elements to coherent series of activities and our concepts of Handlungsspielraum and Creativity in autism have made useful and applicable contributions to the field. Two features of our work limit the possible applicability of methodology in other contexts: first, all our participating children had good language skills and no other significant intellectual disabilities. This was only partly by design: while we had planned to start with this group, we initially hoped to test our approach with children who had more severe communication problems or other comorbidities. However, as a result of the segregative schooling system in Austria this meant engaging an organizational structure and access to children in special schools proved difficult to organize. The second feature was implicit in how the project was envisioned. Although we started to work with the brothers Yvan and Hank in the last stage of the project, our methods are untested in a group context.

We hope to address both of these limitations in our future work. At the time of writing, a new research project has just started that will explore roles for technology in supporting social play in mixed groups of autistic and non-autistic groups.

### 4.3. Outcomes and evaluation

The shift away from explicitly targeting presumed deficits creates a significant challenge to evaluating the outcomes. While classical assistive technologies can be assessed by measuring how efficiently support a disabled person in a certain situation, with technologies like the ones we created here, it is much more difficult to assess whether they have been a success. In Claude’s case for example, his object, Adaja, seemed to be meaningful to him, but he also quickly lost interest and even returned it to us after our collaboration ended. Blaine’s object ThinkM had a very clear use-case scenario, but after our collaboration ended, he did not actually use it. Nevertheless, he placed it in his room, expressed pride and told everyone visiting the story of how he created it during our time together. On the surface, these reports might seem like failures, but looking closer, our assessments produced very nuanced results.

A key prerequisite to getting a grip on assessing the experiences of autistic children with technologies is having a suitable framework that would allow to capture those. In HCI, the concept of experience, and subsequently the idea of experience design (UX), is predominately shaped by the work of Wright and McCarthy (2008). It hinges on the assumption that assessing one’s experience with technology can be captured through an empathetic understanding. For groups, however, who have radically different life-worlds from researchers, such as autistic children, this is more than doubtful. In Spiel, Frauenberger, and Fitzpatrick (2017) and Spiel, Frauenberger, Hornecker, and Fitzpatrick (2017), we address this systemic problem and propose an extended conceptualization of experience that is constructed from multiple perspectives and through diverse data sources. Building on Actor-Network Theory (ANT, Latour (2005)) and Critical Discourse Analysis (CDA, J¨ager and Maier (2009)), we look to qualify the child’s experience from the relations between all actors in the network. While ANT provides us the network of human (e.g., the child, parents, researchers etc.) and nonhuman (e.g., the smart object, software etc.), CDA provides us a way to make statements about the relationships between them. The data sources for such an analysis are manifold, ranging from log data, formal studies to interviews or observations. Two aspects are key here: such an understanding of experience is multi-faceted and might produce contradictory results. For example, the ambiguity of Blaine not using his thinking machine as devised, but telling everyone proudly about its creation becomes part of his experience with the technology.

Another feature of such an extended concept of experience is that it allows room for a perspective that is typically marginalized, particularly with disabled people: the perspective of the people themselves. While self-reports of experiences have obvious limitations, they add a valuable dimension when put into context with others. In Spiel, Malinverni, Good, and Frauenberger (2017), we have made the case for a method for participatory evaluation with autistic children. When facilitating such contributions, they offer unique insights into the overall assessment of how an engagement was successful, or not. In Mia’s case, for example, we invited her to write advertisements for her smart object in a newspaper. The way she portrayed the benefits of having such a device were a valuable contribution in painting a more holistic picture of her overall technology experience.

Participatory Evaluation also opens up a scope for asking more fundamentally which measures of success are relevant. We have argued that this depends on who these measures are for. Involving children’s perspectives in the evaluation can enable researchers to critically reflect on what defines success for different stakeholders in the process. In the case of Mia, the activity around the advertisement prompted her to develop a practice of creating small magazines with stories which she has sustained until the present day and now even distributes copies at school. Being focused on traditional outcome measures would have made us unaware of this success.

While we argue that these approaches are pointing in the right direction, many questions remain open. As above, it is unclear how well these approaches hold up when working with children that have less language or other additional deficits. Certainly, we need innovation in terms of methods to find ways in which this group can effectively take part in co-constructing experience. All the methods we have deployed also require substantial time and effort. It will be necessary to develop methods that are participatory, but more light-weight. Also, we only have looked into a limited range of data sources that we draw on. Different research contexts may open up possibilities to collect very different kinds of data, for which we need to develop new ways of analyzing. It is quite possible that such data comes from more formal user studies which lead to the challenge of how such diverse types of data sources could be meaningfully integrated to make qualifying statements about relationships between actors. Finally, we recognize that we need to disentangle power relationships (compare Bratteteig & Wagner, 2014) in co-constructing experience and critically reflect how these power differences skew our interpretations.

## 5. Conclusion

In this article we have provided the synopsis of the research project “OutsideTheBox Rethinking Assistive Technologies with Autistic Children.” We have laid out the fundamental argument of the project, the aims and planned methodology. We then have presented a series of case studies which tell the stories of our experiences in working with autistic children to create their own smart thing. In the last part of the article, we critically reflect on the project and across the different cases. We do this through three different lenses which correspond to the areas in which this project has aimed to make its main contributions. Arguing for a shift in the *theoretical framing*, we discuss how changing one’s conceptualization of disability leads to being able to imagine very different kinds of technology for disabled people. In *Process*, we reflect on our methodological contributions and their limitations. And finally, in *Outcomes and Evaluation* we discuss how our argument impacts on possible measures of success and their co-construction.

We believe this project has made a difference on several levels. Firstly, we like to think that the participating children have gained from our collaboration and are empowered to think differently about technology. Secondly, we hope that this shift in thinking extends to other stakeholders such as parents, teachers and policy makers. Through various outreach activities, we have also actively sought to carry this discourse into the public domain and kick-start a broader discourse about disability and about what roles we expect technology to take in this context. Academically, we have demonstrated that it is possible to develop smart, digital things in ways that are driven by the ideas of autistic children. We have made significant contributions in terms of theory, methodology and design practice that we hope will be usable for others to build on and extend into other contexts.

## Notes

1. We are aware about complex discussions surrounding person-first vs. label-first language; we opt for the latter, due to it being the predominantly self-chosen form (Kenny et al., 2015).

2. http://igw.tuwien.ac.at/sparkling.

3. Several studies exist that estimate the prevalence of autism spectrum conditions. The results differ depending on context, method and when these studies were conducted (e.g., 1.57% in Baron-Cohen et al. (2009), 2.64% in (Kim et al., 2011), 2.47% in Xu, Strathearn, Liu, and Bao (2018)).

4. German for Scope of Action or literally Action-Play-Space.

5. http://littlebits.cc.
